# Intelligence

**Published:** 1810-02

**Authors:** 


### INTELLIGENCE.

*The* PHYSICAL SOCIETY *of* GUY'S, *have sent the following* CIRCULAR LETTER *to their* HONORARY MEMBERS, *signed by their Secretary.*

"SIR,

"At an especial Meeting of the Committee of the Physical Society, appointed December 22d, 1809, to consider the best means of furthering the general interests of the Society, and more particularly those of the ordinary Members, it was unanimously resolved, that a circular Letter be sent to all the Honorary Members, soliciting their countenance and support. The Members therefore, who regularly attend its Meetings, and who feel warmly for its improvement, request you would favour them by attending as often as you conveniently can; and by frequent communication of physical news, give energy and importance to its debates. By a perseverance in these means they are convinced you could contribute very materially to the improvement of the junior Members."

HENRY HINDE PELLY, Esq. of Upton, Essex, a gentleman advanced in years, and who used to be laid up annually for three or four months, with a violent fit of the gout, having read in some old book, that a loadstone worn next the skin was a sure preservative against that excruciating disease, and knowing that some of the finest and most powerful magnets are found in Golconda, employed an agent in India to procure him one from that province. This stone chipped into a convenient shape, he constantly wears, sewed in a little flannel case, suspended from a black ribbon round his his neck next his skin. It is about two inches long, an inch and a half broad, and two-tenths of an inch thick, and its magnetic virtue is very great. It much resembles a piece of slate, such as school-boys learn to cypher on. Mr. Pelly says, that he now and then has some slight twinges, which only serve to remind him of the terrible paroxysms to which he once was subject. He happened one day to omit hanging this amulet about his neck; another and another day passed, and as several years had elapsed without a fit, he began to think that the magnet had altered his system, and rendered him intangible by gout. One night however he awoke in torment; he called for his safeguard, and threw it about his neck; he escaped with a slight attack, and has never since been without his piece of loadstone, which he wears night and day, and enjoys perfect freedom from all the pains inflicted by his old enemy.

Mr. Clark, Veterinary Surgeon, of Giltspur Street, has for several years paid particular attention to the structure and uses of the several parts of the horse's foot. He has traced its gradual changes from birth through the first five years of the animal's life, when it becomes completely formed; and in the course of this investigation, he has discovered the uses and importance of several parts which were not before known. This has naturally led him to notice the effect of shoeing, a d the changes it produces in the structure and functions of the feet. Hence he has been able to deduce several most important conclusions respecting the preservation of the feet, and the general causes of lameness, particularly in the fore feet, as well as the means of avoiding them. The first part of this interesting work is already printed, and we may expect its publication in a few days.

An American paper contains the following extraordinary instance of depletion, practised on Captain James Niblett, a man thirty years of age, of a full and plethoric habit of body when in health, and accustomed to daily exercise on foot, of a bilious aspect. His complaint was an inflammatory affection of the lungs. From the 28th of May to the 28th of July, Captain Niblett lost, by admeasurement, 600 ounces of blood, and by weight 688 ounces 6 drachms; being, it is presumed, the largest quantity ever drawn from the veins of any human being in the same length of time, by medical advice, and for the person to bear it and do so well. He was bled fifty different times, and the blood every time was covered with a thick, strong, white coat, and lost from four to twenty ounces at each time. He was cupped, and had leeches applied daily, for several weeks, exclusive of the bleedings at the arms, and the discharge from the seton.

The Spring Course of Lectures at St. Thomas's and Guy's Hospitals, commence the beginning of February; viz.

At St. Thomas's, Anatomy, and the Operations of Surgery, by Mr. Cline and Mr. Cooper. Principles and Practice of Surgery by Mr. Co per.

At Guy's Hos ital, Practice of Medicine, by Dr. Babington and Dr. CURRY.—Chemistry, by Dr. BABINGTON, Dr. MARCET, and Mr. ALLEN.—Experimental Philosophy, by Mr. ALLEN.—Theory of Medicine, and Materia Medica, by Dr. CURRY and Dr. CHOLMELEY.—Midwifery, and Diseases of Women and Children, by Dr. HAIGHTON.—Physiology, or Laws of the Animal Œconomy, by Dr. HAIGHTON.—Structure and Diseases of the Teeth, by Mr. FOX.

Dr. HOOPER will commence a Course of Lectures on the Theory and Practice of Physic, the Materia Medica, and Chemistry, on Monday February 5, 1810, at his Lecture Room, in Cork Street, Burlington Gardens, at eight o'clock in the morning. A Prospectus with particulars may be had by applying to Dr. H. at his house, No. 21, Saville Row.

A new work by Dr. MACLEAN will be published in a few days, entitled, "An enquiry into the origin, early signs, nature, causes, and cure of hydrothorax, or dropsy of the chest; in which a safe, speedy, and effectual method of evacuating the water, by combinations, and on principles in many respects new, is pointed out; together with an Appendix, containing a considerable number of interesting cases; and many living testimonies of the superior efficacy of the means recommended.

Mr. ASHFORD, Member of the Royal College of Surgeons, and Assistant Surgeon in the Royal Artillery, has in the press an Epitome of Anatomy, comprised in a series of tables. The work will form a thin quarto volume; and as its object is to furnish a copious vocabulary for the Students of Anatomy, perspicuity and simplicity of arrangement have been chiefly aimed at by the author.

Mr. BENJAMIN TRAVERS, Demonstrator of Anatomy at Guy's Hospital, and Surgeon to the Honourable East India Company, has in the press, and nearly ready for publication, an Experimental Inquiry concerning Injuries to the Canal of the Intestines; illustrating the treatment of penetrating wounds and mortified hernia.

In the course of next month will be published, The New London Pharmacopœia, enlarged from the last Edinburgh and Dublin Pharmacopœias, and reduced to one common Nomenclature; with an Appendix of the Genera, and Species of the different Articles of their Materia Medica. Translated and alphabetically arranged, by RICHARD STOCKER, Apothecary to Guy's Hospital.

